# Supplementary material for: Circulating SPINT1 Is Reduced in a Preeclamptic Cohort with Co-Existing Fetal Growth Restriction
Source: J Clin Med. 2022 Feb 9;11(4):901. doi: 10.3390/jcm11040901 (PMC8877863; doi:10.3390/jcm11040901)
Supplement: Supplementary file 1 [file jcm-11-00901-s001.zip › jcm-1510051-supplementary.pdf]

Supplementary Materials for "Circulating SPINT1 is reduced in a preeclamptic cohort with co-existing fetal growth restriction"

Ciara N. Murphy, Catherine A. Cluver, Susan P. Walker, Emerson Keenan, Roxanne Hastie, Teresa M. MacDonald, Natalie J. Hannan, Fiona C. Brownfoot, Ping Cannon, Stephen Tong and Tu'uhevaha J. Kaitu'u-Lino

Supplementary Table S1: PIE patient characteristics. Samples from pregnancies which culminated in the delivery of an infant with a birthweight centile ≥10th classified as AGA 'controls', and those <10th centile categorised as SGA. BMI data not available for 3 SGA samples.
* $p < 0.05$, ** $p < 0.01$, **** $p < 0.0001$.

		**AGA** n=22	**SGA** n=75
	**gestation at sampling** (days) median (IQR)	206 (198-215)	204 (191-214)
	**gestation at delivery** (days) median (IQR)	236 (215-239)	219 (204-230) **
	**interval between sampling and delivery** (days) median (IQR)	20 (12-33)	14 (6-21) *
	**birth weight** (g) mean (SEM)	1936 (102.7)	1234 (37.8) ****
	**maternal age** (years) mean (SEM)	31 (1.5)	27 (0.7) *
	**BMI** (kg/m^2) median (IQR)	32.5 (27.7-38.0)	28.7 (23.8-33.9) *
**treatment** no. (%)	**placebo**	13 (59)	37 (49)
	**esomeprazole**	9 (41)	38 (51)
**parity** no. (%)	**0**	6 (27)	31 (41)
	**1**	6 (27)	21 (28)
	**≥2**	10 (46)	23 (31)
**blood pressure** median (IQR)	**highest systolic BP** before delivery (mmHg)	156.5 (148.3-159.3)	159.0 (154.0-169.0) *
	**highest diastolic BP** before delivery (mmHg)	101.0 (91.8-104.0)	102.0 (97.0-108.0)
**tobacco use** no. (%)	**smoker**	2 (9)	6 (8)
	**former smoker**	1 (5)	7 (9)
	**non-smoker**	19 (86)	62 (83)
**HIV status** no. (%)	**negative**	16 (73)	64 (85)
	**positive**	6 (27)	11 (15)

Supplementary Table S2: PI-2 patient characteristics. Samples from pregnancies which culminated in the delivery of an infant with a birthweight centile ≥10th classified as AGA 'controls', and those <10th centile categorised as SGA. BMI data not available for 1 SGA sample.
* $p < 0.05$, ** $p < 0.01$, *** $p < 0.001$, **** $p < 0.0001$.

		**AGA** n=40	**SGA** n=95
	**gestation at sampling** (days) median (IQR)	231 (222-233)	219 (205-230) **
	**gestation at delivery** (days) median (IQR)	239 (237-240)	227 (212-239) ****
	**interval between sampling and delivery** (days) median (IQR)	7 (5-10)	5 (4-7) **
	**birth weight** (g) mean (SEM)	2343 (111.1)	1374 (40.84) ****
	**maternal age** (years) mean (SEM)	28.7 (0.6)	31.2 (0.9) *
	**BMI** (kg/m^2) median (IQR)	32.0 (25.8-38.8)	29.4 (25.0-35.5)
**treatment** no. (%)	**placebo**	18 (45)	45 (47)
	**metformin**	22 (55)	50 (53)
**parity** no. (%)	**0**	9 (22.5)	27 (28.4)
	**1**	8 (20)	31 (32.6)
	**≥2**	23 (57.5)	37 (38.9)
**blood pressure** median (IQR)	**highest systolic BP** before delivery (mmHg)	150 (146-159)	159 (152-169) ****
	**highest diastolic BP** before delivery (mmHg)	91 (83-97)	98 (91-101) ***
**tobacco use** no. (%)	**smoker**	5 (12.5)	13 (13.7)
	**former smoker**	1 (2.5)	4 (4.2)
	**non-smoker**	34 (85)	78 (82.1)
**HIV status** no. (%)	**negative**	27 (67.5)	79 (83.2)
	**positive**	13 (32.5)	16 (16.8)
